# Advances in Molecular Imaging of Locally Delivered Targeted Therapeutics for Central Nervous System Tumors

**DOI:** 10.3390/ijms18020351

**Published:** 2017-02-08

**Authors:** Umberto Tosi, Christopher S. Marnell, Raymond Chang, William C. Cho, Richard Ting, Uday B. Maachani, Mark M. Souweidane

**Affiliations:** 1 Department of Neurological Surgery, Weill Cornell Medical College, New York, NY 10065, USA; umt2001@med.cornell.edu (U.T.); csm2002@med.cornell.edu (C.S.M.); rkc2003@med.cornell.edu (R.C.); mmsouwei@med.cornell.edu (M.M.S.); 2 Department of Clinical Oncology, Queen Elizabeth Hospital, Kowloon, Hong Kong, China; chocs@ha.org.hk; 3 Department of Radiology, Molecular Imaging Innovations Institute, Weill Cornell Medicine, New York, NY 10065, USA; rct2001@med.cornell.edu

**Keywords:** central nervous system (CNS) tumors, blood–brain-barrier, convection-enhanced delivery, theranostics

## Abstract

Thanks to the recent advances in the development of chemotherapeutics, the morbidity and mortality of many cancers has decreased significantly. However, compared to oncology in general, the field of neuro-oncology has lagged behind. While new molecularly targeted chemotherapeutics have emerged, the impermeability of the blood–brain barrier (BBB) renders systemic delivery of these clinical agents suboptimal. To circumvent the BBB, novel routes of administration are being applied in the clinic, ranging from intra-arterial infusion and direct infusion into the target tissue (convection enhanced delivery (CED)) to the use of focused ultrasound to temporarily disrupt the BBB. However, the current system depends on a “wait-and-see” approach, whereby drug delivery is deemed successful only when a specific clinical outcome is observed. The shortcomings of this approach are evident, as a failed delivery that needs immediate refinement cannot be observed and corrected. In response to this problem, new theranostic agents, compounds with both imaging and therapeutic potential, are being developed, paving the way for improved and monitored delivery to central nervous system (CNS) malignancies. In this review, we focus on the advances and the challenges to improve early cancer detection, selection of targeted therapy, and evaluation of therapeutic efficacy, brought forth by the development of these new agents.

## 1. Introduction

Neuroimaging and grading of central nervous system (CNS) and, specifically, brain tumors has evolved from a purely anatomy-based discipline as per the World Health Organization (WHO) classification (which merely relied on the basis of histological features and almost entirely on microscopic visual assessment of tumor bio-specimen) [1] to one that incorporates morphologic abnormality with physiologic alterations in extracellular compartment kinetics, cellular metabolism, and hemodynamics [2]. The WHO classification scheme of brain tumors remains the primary basis for managing therapy and evaluating overall prognosis in patients with brain tumors. The current diagnostic approaches fall short of predicting therapeutic response of individual tumors and cannot provide precise guidance for therapy, especially for those targeting specific molecular or genetic pathways of tumorigenesis [3]. Despite these shortcomings, the most recent WHO classification (2016 revision) started taking into account these molecular mechanisms. This work, however, is still at a germinal phase.

**Figure 1:**
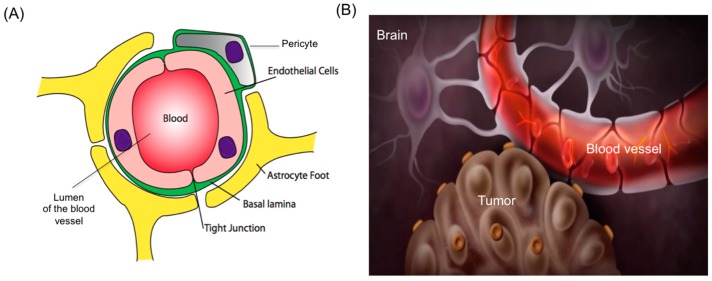
(**A**) Schematic illustration of the blood–brain barrier in cross section maintained by brain capillary endothelial cells and astrocytes via tight junctions; (**B**) Brain architecture illustration along with blood vessels and tumor.

Recent advances in molecular neuro-oncology provide unique opportunities for targeted molecular-based therapies. However, the major challenge to the delivery of both therapeutic and molecular imaging agents is transportation across the blood-brain barrier (BBB) [4]. The BBB is maintained by brain capillary endothelial cells (BCECs) and astrocytes via tight junctions (as shown in Figure 1), is a selectively permeable barrier that protects the brain from foreign substances, facilitates the uptake of nutrients into brain parenchyma, and transports metabolites and toxins away from the brain.

While small nonpolar agents are transported passively through the BBB, polar molecules or those over 400 Da in size require active transport to reach the brain [4,5]. The presence of efflux transporters in the BBB that commonly act on chemotherapeutics (e.g., P-glycoprotein) also further limits drug delivery to CNS tumors [6,7]. A promising avenue towards improving delivery of theranostics (agents with both imaging and therapeutic potential) to the CNS relies on transient disruption of the BBB. The advantage of such an approach over specifically designing agents that cross the BBB is that the former can accommodate a variety of molecular imaging and chemotherapeutic agents that have proven effective for non-CNS neoplasms, but may have poor CNS penetration when administered systemically. Clearly, there is a need for improved delivery and imaging that can guide therapy and assess early treatment response, eventually indicating clinical outcomes and measures. In this review, we focus on the approaches and the clinical application of these methods to patients with brain tumors.

## 2. Strategies for CNS Delivery

Numerous different strategies are being undertaken to bypass the tightly-regulated BBB. Here, we present those that we find of most importance to clinical practice, thus leaving certain techniques undiscussed. Generally, the choice of one methodology against the other depends on the clinical picture and on the agent that needs delivering. For instance, CED is highly effective at delivering high regional concentrations of both large and small molecules; osmotic agents allow for a more widespread distribution, at a cost of a potentially lower regional concentration. As such, it is in the hands of the clinician to choose the most appropriate delivery method for each situation.

### 2.1. Focused Ultrasound (FUS)

Focused ultrasound (FUS) disruption of the BBB is emerging as a novel strategy for enhanced delivery of therapeutics into the brain via focal, reversible and safe BBB disruption [8]. Recent iterations of FUS utilize low-frequency ultrasound waves coupled with injection of lipid- or polymer-based microbubbles into vasculature to disrupt the BBB [9,10]. Contrast-enhanced magnetic resonance imaging (MRI) may be used to guide FUS and as a possible indicator of drug penetration [11,12]. Microbubbles oscillate in response to the cyclical pressure changes when traveling through tissue targeted by FUS and may mechanically disrupt tight junctions in the BBB [13,14]. Microbubbles enable this disruption at low mechanical indices potentially due to such mechanisms as stable cavitation (oscillation of microbubble), inertial cavitation (collapse and jetting of fluid), microstreaming (fluid flow generated around oscillating microbubbles), and even translation of the bubble across vessel walls [15,16,17]. Further disruption of the tight junctions allows paracellular passage of substances of macromolecules, with effects lasting up to 4 h after ultrasound application [14]. Barrier function and protein expression levels of tight junctions in the BBB appear to be restored at 6–12 h post-application, suggesting that disruption (and thus delivery) is temporary [13,14]. Moreover, FUS appears to transiently enhance the permeability of the blood-brain-tumor barrier (BBTB) as well [18,19]. Lastly, in addition to enhancing passive, paracellular passage through the BBB, there is evidence that FUS may increase active transport of substances up to 4.95 MDa across the cell membranes through active vesicular transport, though one report suggested that delivery (of adeno-associated virus, in this case) into cell cytosol was not due to endocytosis [20,21,22,23,24]. In terms of actual treatment agent delivery, multiple preclinical studies have assessed enhancing the delivery of chemotherapy into the brain with FUS [12,25,26]. FUS has also been demonstrated to increase delivery of antibodies in to the brain, significantly reducing plaque in a transgenic mouse model of Alzheimer’s disease [27]. Finally, recent work suggested that sub-micrometer, nanobubbles (as opposed to supramicrometer microbubbles) may have applications for FUS as well. While nanobubbles have lower ultrasound scattering efficiency than microbubbles, they can penetrate disrupted tumor vascular beds, can be more stable than microbubbles, and are also less likely to undergo inertial cavitation and cause micro hemorrhages [28,29,30,31,32]. A study by Huang et al. [28] demonstrated that magnetically guidable nanobubbles can disrupt the BBB and serve as a contrast-enhancing agent for ultrasound (US) and MRI, while causing a lower rate of erythrocyte extravasation than their supra-micrometer sizes counterparts or a commercially available, lipid-based microbubble [28]. In summary, exciting new advances in FUS have the potential to expand both imaging and drug delivery capabilities in the treatment of CNS tumors.

### 2.2. Osmotic Agents

Another avenue of transient BBB disruption (BBBD) utilizes intra-arterial infusion of osmotic agents. Hyperosmotic solutions like mannitol cause shrinkage of BCECs and local vasodilatation, enabling paracellular movement of substances through the BBB, increasing permeability by both increased diffusion and bulk fluid flow [33,34,35,36,37,38,39,40,41,42,43,44]. Clinical trials utilizing osmotic BBBD have demonstrated increased survival or radiographic responses in malignant gliomas, CNS lymphoma, and brain metastases as compared to standard therapeutic modalities [36,37,40,41,43]. Fortin et al. observed increased mean survival times (MSTs) for ovarian carcinoma, lymphoma, and lung carcinoma brain metastases as compared to reported median survival [41]. Hall et al. reported longer median time to tumor progression (15 months) and MST (27 months) than previously reported for a series of eight diffuse intrinsic pontine glioma (DIPG) patients treated with osmotic BBBD and chemotherapy [45]. Finally, beyond chemotherapeutics, several clinical trials have used osmotic BBBD to deliver antibodies (particularly bevacizumab) to treat recurrent malignant gliomas [36,37,43]. Disadvantages of the osmotic agent approach include the fact that such disruption is inherently non-selective and may lead to toxic metabolites passing into the brain, and such disruption will be spatially constrained by the distance osmotic agents can travel from the internal carotid artery before losing their BBBD effects [46,47].

### 2.3. Receptor-Mediated Agents

Recent discoveries in vascular biology identified several molecular agents that take advantage of receptor-mediated mechanisms of enhancing BBB permeability. Two notable examples include bradykinin analog RMP-7 and calcium-dependent potassium channels (KCa) [48]. RMP-7 is a bradykinin B2 receptor agonist with an increased half-life over bradykinin. Stimulation of B2 receptors on BCECs increases tight junction permeability and allows for paracellular penetration of the BBB [49,50]. Preclinical models demonstrated that RMP-7 enhanced carboplatin passage through the BBB when administered either intra-arterially or intravenously [51]. However, clinical trials failed to show any benefit in pediatric brain tumors and recurrent malignant gliomas [52,53]. Prados et al. suggested that higher dosing (1200–1500 ng/kg) timed to coincide with the C_max_ of carboplatin may be necessary to increase levels of carboplatin reaching the brain [52]. Studies show that KCa channels in cerebral blood vessels can regulate tone and possibly BBB permeability [54]. KCa channels may also play a part in the vasodilation mediated by bradykinin [55,56]. Preclinical work has suggested that brain tumor and brain tumor capillaries overexpress KCa, potentially providing an avenue for blood-brain-tumor barrier-specific disruption [57].

### 2.4. Convection Enhanced Delivery (CED)

Despite the current efforts to bypass the BBB via systemic delivery, a different route of administration has risen that reshapes the problem, eliminating the need for crossing the tight BBB. This technique originated in the 1990s [58], with the first successful clinical trial performed in 1997 by the Oldfield group on glioma patients, where a significant tumor regression was observed in the majority of patients [59].

**Figure 2:**
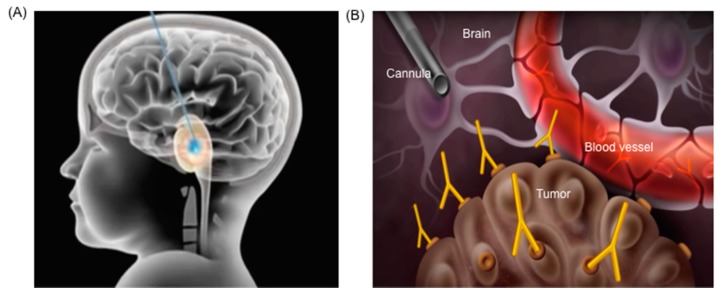
(**A**) Schematic illustration of a cannula implanted for convection-enhanced delivery (CED) in brain stem; (**B**) Illustration of cannula implanted stereotactically in proximity of the target of interest in the brain for delivery of therapeutics.

Convection enhanced delivery (CED) is a technique based on a cannula implantation and delivery via a pressure gradient of a therapeutic of choice [60]. A cannula is implanted stereotactically, with its tip in proximity (or in the center) of the target of interest (Figure 2).

**Figure 3:**
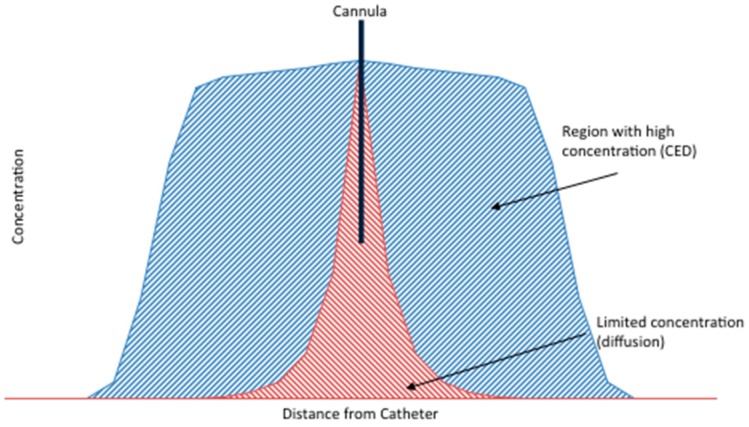
Graphic depiction comparing the distribution associated with diffusion away from a single point and “bulk flow”, based on a pressure gradient, as obtained in convection enhanced delivery (CED) of brain.

An injector is then used to allow for a constant rate of infusion [61,62]. The technique relies on “bulk flow”: tissue permeation does not depend on the physical properties of the infusate but, rather, is determined by the infusion pressure, rate, and intrinsic tissue properties. As such, a steep gradient rather than an exponential one is established, allowing for both deep penetration into tissue and high concentration gradients [63]. As shown in Figure 3, this is superior to regular diffusion, where an exponential decrease in concentration is observed as a function of distance. Following delivery (which can take from minutes to, more commonly, hours) the cannula is removed. New approaches are now being investigated to allow for multiple deliveries to achieve higher or more prolonged regional concentrations of therapeutics. These rely on either multiple surgical interventions or on implantable devices (such as pumps connected to a pre-implanted catheter) that allow for longer infusion times [64,65].

CED allows obtaining local high concentrations of the drug that are dependent on tissue properties rather than drug ones, hence allowing uniformity across different therapeutics (bulk flow, unlike diffusion, does not depend on molecular weight) [66]. Over time, the sharp edge of the pressure function decreases, as an exponential diffusion function away from injection point is superimposed on it [60,67]. Despite the theoretical simplicity of the technique, numerous concrete challenges remain and contributed to the initial failure of the procedure. Firstly, the diffusion rate is dependent on tissue properties. As such, white matter tracts are a low resistance pathway to convection, potentially behaving as a “sink”, removing infusate from the higher density grey matter [66]. Certain densely packed brain regions, such as the pons, present even lower perfusion rates, and initially made the technique unsuccessful [62,68]. Secondly, backflow is a significant restriction to the ability to entirely perfuse the region of interest, as flow along the cannula tract reduces the pressure at the infusate front, hence reducing its distribution through the parenchyma. Furthermore, backflow poses the risk of significant complications such as chemical meningitis [69]. Backflow is directly proportional to both catheter size and infusion rate. Recent studies showed that up to 30% of the infused volume were “lost” because of backflow [70]. To overcome this issue, significant effort has been invested into catheter design—for instance, studies suggest that the use of a STEP catheter (where a smaller cannula is used as the tip of a slightly larger needle) can significantly reduce the amount of backflow, without, however, completely eliminating it [71]. Larger catheters create a low-resistance tract on the catheter surface, thus favoring backflow. Similarly, the infusion rate can be reduced by concentrating the infusate—this, however, lengthens the procedure with potential for complications. Furthermore, backflow depends on tissue density as well, making effective delivery more complex in denser tissues like the pons [66]. A good balance has to be obtained between reducing flow rate to diminish backflow and increasing it to maximize the distribution volume (V_d_), which increases with infusion rate because of higher velocities at the cannula tip. Recent studies have analyzed different tissue properties in rodent models in an effort to find the ideal balance [66]; a careful characterization in clinical patients, however, is still missing.

Lastly, a major setback of CED is due to the potentially different physical properties of the target tissue. For instance, numerous CNS malignancies do not have constant density—especially if prone to cystic lesions or necrosis. As such, equal delivery through the target tissue becomes impossible—similarly, assessing the efficacy of the therapeutic of choice becomes arduous, and is currently the focus of different research endeavors. If constant drug concentration in tissue cannot be achieved, it is impossible to determine whether such a drug works against the malignancy [62].

To overcome the issue of tissue dependent volume of distribution, significant efforts have been undertaken in the design of new, ingenious catheters. These, placed under MRI guidance to confirm proper targeting [72], include STEP catheters, with progressively smaller ends to allow for smooth infusion with limited backflow [73]; the use of multiple catheters with potentially different infusion parameters, thus accounting for variable tissue properties [74], and the use of multi-port catheters [75] that allow to more carefully sculpt delivery. Albeit numerous studies are being performed in this investigative direction, many more will be needed, as behavior of the injected compounds is hard to predict and model beforehand.

The number of clinical trials relying on CED is scarce and, as such, a thorough understanding of the risks involved in the procedure is still ongoing. However, the evidence available indicates that chemical meningitis, infection at surgical site, and transient neurological deterioration are all possible complications of CED. The rate at which these occur is still unclear and requires further investigation [76,77,78]. It is important to highlight how CED is often used as a last-resort method in patients for whom systemic chemotherapy or other safer treatments have failed. This has to be taken into account when a risk–benefit analysis is carried out.

Overall, CED proved to be an ingenious solution to BBB permeability problem, allowing for high concentrations of therapeutic to be focused on a brain region of interest. Numerous challenges, however, remain.

### 2.5. Advances in Imaging and Theranostics

The advent of new techniques, of which CED seems the most promising, made it clear that the BBB can be surpassed, thus allowing for drug delivery into the brain [61,62]. A problem, however, remains. The current method to determine whether a delivery in the brain has been successful relies on a “wait-and-see” approach, whereby a procedure is deemed successful only after a clinical outcome is observed. The shortcomings of this method are evident: in case a delivery is unsuccessful (say, a sufficient concentration of infusate is not reached in the target tissue), no room for correction is present until a clinical outcome fails to develop—at times, weeks after the procedure [79].

To solve this issue, initial efforts were focused on the co-infusion of the therapeutic of choice with an imaging agent (most notably, the MRI tracer gadolinium). Initial studies showed how gadolinium was a sufficient proxy that allowed for V_d_ approximation, since the volume of distribution of CED does not depend on infusate properties but rather on physical properties of target tissue and on the infusion rate and cannula size [80,81]. Even though gadolinium and other coinfused imaging agents were sufficient in determining the V_d_ (basically determining where in the brain the infusate went), they failed in assessing the clearance rate. Clearance rate, unlike V_d_, which depends on bulk flow, is a property intrinsic to a molecule, and a tracer with different properties cannot correctly represent it [80,82,83].

Given these problems, a new class of molecules was developed that could accurately predict a volume of distribution and clearance rate: theranostics [84,85]. A theranostic agent is a molecule with both therapeutic and imaging properties, based on the assumption that the best imaging proxy for a therapeutic agent is the agent itself (or its closest representation) [86,87]. Importantly, theranostics can be visualized with more than one imaging modality; for instance, the high sensitivity of positron emission tomography (PET) is coupled with the spatial resolution of computed tomography (CT) or MRI, thus obtaining optimal imaging. Recent efforts also focused in the discovery of single agents that can be imaged by more than one modality, thus coupling the advantages of each one. For instance, melanin, whose levels are altered in some melanomas, can be observed via MRI (because of its high iron binding activity) [88], PET (thanks to ^18^F probes synthesized for the purpose) [89], and photoacoustic imaging (PAI) due to its wide absorption range [90,91]. These multimodal approaches allow the researcher and, potentially, the clinician to obtain a broader picture that will influence therapy.

New theranostics are being developed constantly and, presently, they can be broadly divided into three distinct classes: antibody carries, nanocarriers, and labeled small molecules, as exemplified below. Each of these has its own merits and downfalls, and deserves a more thorough analysis.

#### 2.5.1. Antibody Carriers

In the simplest configuration, monoclonal antibodies against a therapeutic target are synthesized and coupled with a radioisotope (most commonly, ^124^I and ^131^I), thus having both therapeutic and imaging properties. Bevacizumab is a common anti-VEGF (vascular endothelial growth factor) antibody used in numerous cancers to contrast tumor angiogenesis, including glioblastoma multiforme (GBM) as a single agent [92]. The agent has been successfully coupled with ^89^Zr, a PET-imageable atom, thus allowing clinicians to observe tumor uptake following delivery [93]. Our laboratory has experience with the synthesis of antibodies against the cancer-specific antigen B7-H3 and their coupling with ^124^I—in this setup, the imaging agent (the radioactive iodide, detected with PET) is also the therapeutic one, with isotope decay being the main method of injury to cancer cells [83]. Such a design allows for careful determination of dosimetry and V_d_ and, at the same time, showed promising therapeutic potential. Importantly, numerous other tumor antigens can be targeted.

Other, more complex systems depend on the antibody-antigen interaction to deliver a chemotherapeutic, thus obtaining a high level of cell-specificity. A different site of the antibody is then used for conjugation of a radiotracer, thus obtaining a three-component system (drug-antibody-isotope) [94]. Importantly, these compounds can be delivered both systemically or locally in the brain parenchyma, depending on where the target is located. Given that the BBB is mostly impermeable to such constructs, CED may be necessary. This method, however, heavily relies on the ability to conjugate a drug to the immunoglobulin without losing its therapeutic potential. Hence, only certain drugs that have a readily accessible functional group for coupling as far away from the drug active site as possible can be utilized. Recent efforts showed success with the small molecule kinase inhibitor dasatinib, but other compounds may prove more challenging [94]. One major drawback of this methodology, however, exists. Different studies have shown how the infusion of antibodies can cause a rapid development of an immune response, whereby the patient’s immune system effectively neutralizes the infused antibodies (especially if from a different species, as it is the case for most of the agents considered). These studies, conducted initially with transplant patients, showed a rapid dampening of the initially strong response [95,96,97]. Further studies are necessary to determine whether this immune-mediated dampening of a response is of significance even with the use of theranostic complexes. If so, a steer towards human-derived immunoglobulin will be necessary.

#### 2.5.2. Nanocarriers

Nanocarriers represent a broad class that includes different types of nanometer-size molecules on which a therapeutic agent of choice is loaded. These allow for the concomitant transport of more than one drug of choice along with an imaging agent. Different types of nanocarriers exist, including liposomes, nanospheres, nanofibers, carbon microtubules, and others [66,84,98,99,100]. Each is characterized by specific properties that ultimately determine their therapeutic potential. Their characterization is so vast that it by far exceeds the scope of this review and should be addressed elsewhere [66,101,102,103,104]; briefly, specific properties determine tissue penetration (including ability to bypass the BBB), loading/unloading potential, and imaging possibility. Theranostic nanocarriers can represent a three-part system composed of the carrier itself, the loaded therapeutic(s), and the imaging agent [105,106,107], or two part system where carrier is also an imaging agent (for e.g., superparamagnetic iron oxide nanoparticles; these agents, by virtue of the molecular structure containing paramagnetic iron oxide, are MRI-active agents and, as such, do not need coupling with other tracers, constituting a bivalent system [108]).

A second use of nanocarriers involves their utilization for direct tumor ablation [109,110]. Photo- or magneto-sensitive nanoparticles are delivered at tumor site and activated via radiation whose wavelength depends on the specific carrier. For instance, gold nanoparticles can generate heat following exposure to near-infrared (650–950 nm) light [30,111,112]. Superparamagnetic particles, on the other hand, can resonate following exposure to alternating magnetic fields—resulting in localized heat production [113,114,115,116]. Studies on the feasibility of these technologies in the clinical realm are ongoing [117]. These nanocarriers could also be used as imaging agents (theranostics)—for instance, magneto-sensitive platforms are rather apt for such a role, given the ability to image them with MRI.

Different challenges exist in the field of nanometer-size transporters, ranging from successful loading of a therapeutic drug to meaningful and controlled unloading [100]. The three-component system also represents a suboptimal vector for drug imaging, as the loaded drug and imaging agent could be uncoupled at different times or in different regions in the target tissue. Imaging could thus be conjured, without visualizing where the drug is distributed, and with no insight on the therapeutic agent. These shortcomings are more chemical in nature and require close collaboration between the laboratory and the clinic to be resolved.

#### 2.5.3. Labeled Small Molecules

The last class of theranostic agents is composed of labeled small molecular therapeutics [118,119]. In the simplest of form, a chemotherapeutic contains, as part of its molecular structure, a probe with imaging potential. This is usually added a posteriori to a drug with elevated therapeutic potential [120,121], rather than be an intrinsic constituent of a therapeutic (albeit molecules containing metal moieties do have some imaging potential of their own). The addition of an imaging probe requires it not influencing the drug distribution or therapeutic property significantly [122]. Different probes have been tested, but, thus far, the bulkiness of most of them made them unsuitable, significantly altering the properties of small molecules. Our laboratory has experience with small F_3_B-group PET probes to generate coupled small molecules [123,124,125,126]. These fluoride probes are generated by ^18^F–^19^F isotope exchange on one of the fluoride atoms, thus transforming the added group into a PET probe that can be easily imaged [127,128]. This technique allows for the addition of a small group that does not significantly alter molecular properties—when conjugated away from the active site of the molecule. Other efforts focused on different compounds, such as the 5-HT_2A/2C_ antagonist FECIMBI-36, coupled with a single ^18^F atom to obtain a PET theranostic [129]. A similarly created molecule (Cimbi36, a 5-HT_2A_ agonist) coupled with ^11^C has also being used in humans to assess serotonin receptor levels [130]. However, our experience also suggests that some behavior is indeed influenced by the addition of our F_3_B- group or imaging atoms. For instance, a few synthesized molecules showed a significant loss of bioactivity against numerous cancer cell lines and, as such, were not analyzed in vivo. A careful in vitro analysis of the synthesized compound is necessary to guarantee that efficacy is maintained.

Overall, the use of labeled small molecules is the one that more closely adheres to the definition of a theranostic. At the same time, however, the chemistry necessary for the creation of such compounds remains challenging: a new research avenue has been opened up for further investigation.

## 3. Challenges for Targeted Therapeutic Agents of Brain Tumors

Despite extensive basic research and numerous clinical trials, high-grade gliomas remain among the deadliest forms of cancer for both adults and children [131,132]. In adults, glioblastoma multiforme (GBM) is the most common primary malignant CNS tumor, and carries a poor prognosis. Similarly, in children, diffuse intrinsic pontine glioma (DIPG) represents a childhood CNS cancer with a uniformly lethal prognosis, with no significant improvement in survival having occurred in over thirty years of research [133]. While WHO classifications of brain tumors have traditionally emphasized histopathological characteristics, the 2016 WHO classification placed molecular characteristics at the forefront, introducing new classifications like isocitrate dehydrogenase (IDH) wild-type and IDH mutant glioblastomas, and H3 K27M mutant diffuse midline gliomas [133,134]. With increasing insight into the molecular characteristics of brain tumors and new classifications according to genomic analyses, identifying what actionable alterations exist is of paramount importance in guiding precision therapy.

An exhaustive discussion of targeted therapies for CNS malignancies is beyond the scope of this review. Key pathways are summarized herein for which molecular imaging, local delivery and development of theranostic targeted therapies may be useful (Table 1).

## 4. Conclusions

CNS malignancy remains a challenge in management of disease progression in both adults and children. Evaluating the efficacy of new treatment paradigms is extremely laborious and expensive owing to the standard clinical end points of radiographic response and survival outcomes. Evidently, there is an unmet need to improve the delivery of drugs and imaging modalities that can accurately measure therapeutic responses.

The failure of many sophisticated conventional treatments (surgery and radiotherapy) to control high-grade brain tumors necessitates the development of new therapeutic paradigms. Advances in molecular biology and new developments in imaging techniques position targeted therapeutics to play a major role in CNS disease management. Further, the delivery of drugs and macromolecules to the brain following systemic, intravascular administration is hindered by the existence of the BBB [4,5], a hurdle that must be overcome to achieve adequate therapeutic concentrations. The heterogeneity of high-grade brain tumors [3] and the unique environment of the CNS also affect the drug distribution. All these hindrances have led investigators to explore local and regional routes of administration of conventional and new therapeutic agents [62]. Many approaches are being explored to enhance the delivery of these agents across the intact BBB. As discussed in this review, each has the potential to play a significant role in the treatment of CNS disease, but accurately assessing delivery and measuring therapeutic response is still lacking. Theranostic neuro-imaging strategies offer exciting potential to monitor disease progression and defining the challenges in translating and optimizing drug delivery to CNS tumors to improve clinical outcomes.

In summary, this article discusses the development and application of new theranostic agents, that encompass properties of both imaging and therapeutic agents, and may ultimately guide therapeutic decision-making and inform the design of future translational brain tumor studies.

## Figures and Tables

**Table 1:** Current molecularly targeted molecular therapies under development and in ongoing clinical trials for central nervous system (CNS) tumors, highlighting attempts at theranostics and local delivery in the clinical setting.

Agent	Mechanisms	Clinical Trials	Theranostics/Local Delivery
*Angiogenesis Inhibitors*			
VEGF mAbs Bevacizumab	Inhibits vascular endothelial growth factor A (VEGF-A) [135]	No effect on OS in recurrent GBM [135,136,137,138]	^111^In-bevacizumab SPECT in melanoma, RCC and CRC [139,140,141]; ^89^Zr-bevacizumab PET in primary BC [142,143]; Intra-arterial delivery of bevacizumab [37]
*Epigenetic Therapies*			
HDAC inhibitors Panobinostat Vorinostat	Restore histone acetylation in histone mutated gliomas (GBM [144,145], medulloblastoma [146,147] and DIPG [148,149,150,151])	Single agents in GBM [152] and DIPG [NCT02717455]; Combined agents [153]; Radiosensitizers [154]	-
*Growth Factor Signaling*			
EGFR mAbs Cetuximab ABT-414	Block EGFR signaling via binding extracellular domain. ABT-414 is an antibody-drug conjugate targeting EGFR/EGFRvIII	Cetuximab + temozolomide + XRT [155,156]; ABT-414: Phase II [NCT02573324]	^123^I cetuximab crosses BBB, accumulates in NSCLC brain metastases [157]; Cetuximab SSIACI + mannitol BBBD [158]
EGFR TKIs Erlotinib Gefitinib	Block intracellular tyrosine kinase activity of EGFR	Limited single agent effect in Phase II studies; toxicities leading to early termination [159,160,161,162,163]	-
PI3K/mTOR inhibitors Everolimus Tacrolimus Sirolimus	Blockade of PI3K/mTOR growth signaling pathways	Everolimus + TMZ + XRT shows PET-visualized antiproliferative effects in GBM [164]; Everolimus in DIPG [NCT02233049]	-
PDGF/PDGFR Dasatinib Vandetanib	Targets PDGFR signaling; *PDGFRA* amplifications common in both adult and pediatric high-grade gliomas [165]	Dasatinib in DIPG [NCT02233049, NCT01644773]; Vandetanib in GBM shows no change in OS [166]	-
*Immunotherapy/Vaccines*			
Vaccines Rindopepimut SL-701	Vaccines establish immune response to either mutant EGFRvIIII antigen (rindopepimut) [167] or IL-13Ra2, survivin, and Epha2 (SL-701); additional personalized tumor lysate vaccines are under development	Rindopepimut + GM-CSF in newly diagnosed GBM patient prolongs PFS and OS with minimal toxicity [168]; Phase III discontinued [NCT01480479]; SL-701 in Phase I/II for GBM [NCT02078648]; BTIC/Imiquimod in DIPG [NCT01400672]	-
Checkpoint Inhibitors Ipilimumab Nivolumab	mAbs which target either CTLA-4 (ipilimumab) or PD-1 (nivolumab) enhancing immune system antitumoral response [169]	Phase III: Nivolumab + ipilimumab in recurrent GBM [NCT02017717]; Nivolumab in new GBM [NCT02617589]	-
Cell-based Therapies CAR-T	Chimeric antigen receptor transduced peripheral blood lymphocytes initiate cell-mediated cytotoxicity of target cells (i.e. against EGFRvIII) [170]	Phase I/II: GBM [NCT01454596]	-
*Other*			
124I-8H9	MAb 8H9 recognizes B7-H3, extracellular antigen [83]	Phase I: DIPG [NCT01502917]	Agent delivered via CED

HDAC: histone deacetylase; EGFR: epidermal growth factor recepton; XRT: radiotherapy; NSCLC: non-small cell lung cancer; SSIACI: superselective intraarterial cerebral infusion; TKI: tyrosine kinase inhibitor; TMZ: temozolomide; PI3K: phosphoinositide 3-kinase; mTOR: mechanistic target of rapamycin; PDGFR: platelet-derived growth factor receptor; OS: overall survival; IL-13Ra2: interleukin-13 receptor subunit alpha-2; Epha2: Ephrin type-A receptor 2.
